# Activity-Based Detection and Bioanalytical Confirmation of a Fatal Carfentanil Intoxication

**DOI:** 10.3389/fphar.2018.00486

**Published:** 2018-05-15

**Authors:** Annelies Cannaert, Lars Ambach, Peter Blanckaert, Christophe P. Stove

**Affiliations:** ^1^Laboratory of Toxicology, Department of Bioanalysis, Faculty of Pharmaceutical Sciences, Ghent University, Ghent, Belgium; ^2^Laboratory of Toxicology, National Institute of Criminalistics and Criminology, Brussels, Belgium; ^3^Belgian Early Warning System on Drugs, Drugs Program, Scientific Institute of Public Health, Brussels, Belgium

**Keywords:** synthetic opioids, untargeted screening, activity-based, bioassay, carfentanil, LC–MS/MS

## Abstract

Carfentanil, one of the most potent opioids known, has recently been reported as a contaminant in street heroin in the United States and Europe, and is associated with an increased number of life-threatening emergency department admissions and deaths. Here, we report on the application of a novel *in vitro* opioid activity reporter assay and a sensitive bioanalytical assay in the context of a fatal carfentanil intoxication, revealing the highest carfentanil concentrations reported until now. A 21-year-old male was found dead at home with a note stating that he had taken carfentanil with suicidal intentions. A foil bag and plastic bag labeled “C.50” were found at the scene. These bags were similar to a sample obtained by the Belgian Early Warning System on Drugs from a German darknet shop and to those found in the context of a fatality in Norway. Blood, urine and vitreous, obtained during autopsy, were screened with a newly developed *in vitro* opioid activity reporter assay able to detect compounds based on their μ-opioid receptor activity rather than their chemical structure. All extracts showed strong opioid activity. Results were confirmed by a bioanalytical assay, which revealed extremely high concentrations for carfentanil and norcarfentanil. It should be noted that carfentanil concentrations are typically in pg/mL, but here they were 92 ng/mL in blood, 2.8 ng/mL in urine, and 23 ng/mL in vitreous. The blood and vitreous contained 0.532 and 0.300 ng/mL norcarfentanil, respectively. No norcarfentanil was detected in urine. This is the first report where a novel activity-based opioid screening assay was successfully deployed in a forensic case. Confirmation and quantification using a validated bioanalytical procedure revealed the, to our knowledge, highest carfentanil concentrations reported in humans so far.

## Introduction

Carfentanil, a very potent derivative of the pharmaceutical opioid fentanyl, was developed in 1974 by Janssen Pharmaceutica (Van Bever et al., 1976). It is one of the most potent opioids known at ∼10,000 times the potency of morphine and ∼30–100 times the potency of fentanyl in the tail withdrawal test in rats (Van Bever et al., 1976). Commercially, it is always sold in combination with the μ-opioid antagonist naloxone due to its extreme toxicity in humans. Carfentanil is used to immobilize large exotic wildlife and has been implicated in the 2002 Moscow theater hostage crisis (Wax et al., 2003; Riches et al., 2012). Recently, carfentanil and other synthetic opioids have been reported as a contaminant in street heroin in the United States and Europe, and have been associated with an increased number of life-threatening emergency department admissions and deaths (EMCDDA and Europol, 2017; Papsun et al., 2017; Shanks and Behonick, 2017; Shulman et al., 2017). Here, we report on the application of a novel cell-based bioassay and a sensitive bioanalytical assay in the context of a fatal carfentanil intoxication, in which we found the highest carfentanil concentrations reported until now.

## Case Presentation

**FIGURE 1:**
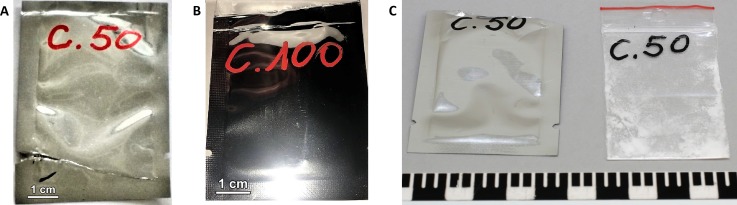
**(A)** Foil bag found at the scene. **(B)** Foil bag obtained by the Belgian Early Warning System on Drugs. **(C)** Foil bag and plastic bag found in a fatality in Norway [image used with kind permission of the National Criminal Investigation Service/Photo (Norway)] (Vevelstad and Drange, 2017).

A 21-year-old male was found dead at home along with a note stating that he had taken carfentanil with suicidal intentions, in addition notifying first responders that care should be taken, given the potency of the compound. A foil bag and plastic bag labeled “C.50” were found at the scene (**Figure 1A**), suggesting that up to 50 mg of carfentanil may have been insufflated by the decedent. Remarkably, during routine monitoring of new psychoactive substances (NPSs) present on darknet websites by the Belgian Early Warning System on Drugs, a carfentanil sample was obtained with strikingly similar packaging and handwriting as the packaging found on the scene of death in this toxicological case (**Figure 1B**). A similar bag with identical labeling in similar handwriting has also been reported in the context of a fatality in Norway (**Figure 1C**), where the powder was apparently ordered from a German darknet shop (Vevelstad and Drange, 2017). Based on this information, the vendor (or primary source) is most probably the same vendor as mentioned in other publications (Marlin and Hoyte, 2017; Vevelstad and Drange, 2017).

A swab of the plastic bag tested positive for carfentanil via GC–MS analysis. Biological matrices available were blood, urine and vitreous. Routine toxicological analyses were performed on peripheral blood and urine. This involved, in addition to immunological screening by EMIT and ELISA, the use of HPLC-diode-array detection (DAD) and GC–MS for screening and quantification of drugs and headspace-GC-FID for the determination of ethanol and other volatile compounds, essentially following procedures described before (Stove et al., 2013). GC–MS screening of blood and urine revealed the presence of caffeine, theobromine, propranolol, sertraline, and cannabinoids in non-toxic doses. Immuno-assay based screening for fentanyl (Fentanyl Direct Elisa Kit, Immunalysis, Pomona, CA, United States) was negative.

An additional opioid screening of the biological matrices was done with a new in-house developed opioid activity reporter assay. We recently reported on cell-based cannabinoid reporter assays for the activity-based detection of synthetic cannabinoids and their metabolites, demonstrating cannabinoid activity in authentic urine and blood samples (Cannaert et al., 2017). A similar bioassay using the μ-opioid receptor to screen for opioid activity in bulk materials and biological samples was set up and evaluated (Cannaert et al., 2018). The principle of the bioassay is activity-based, using an *in vitro* cell system, in which activation of the μ-opioid receptor leads to the recruitment of the cytosolic β-arrestin 2 (βarr2) protein, which results in functional complementation of a split NanoLuc luciferase, thereby restoring luciferase activity. In the presence of the substrate furimazine, this results in a bioluminescent signal, which can be read out with a standard luminometer.

In practice, expression vectors encoding human μ-opioid receptor or βarr2, fused via a flexible linker to the subunits of NanoLuc luciferase (LgBiT or SmBiT), were generated using standard molecular biology techniques, similar as in Cannaert et al. (2016). These constructs, with addition of a G-protein coupled receptor kinase 2, were used to transiently transfect human embryonic kidney (HEK) 293T cells, which were seeded in poly-D-lysine-coated 96-well plates at 5 × 10^4^ cells/well and incubated overnight before performing the assay. On the day of the assay, the cells were washed twice with Opti-MEM^®^ I reduced serum medium to remove any remaining fetal bovine serum, and 90 μL of Opti-MEM^®^ I was added. The Nano-Glo Live Cell reagent, a non-lytic detection reagent containing the cell-permeable furimazine substrate, was prepared by diluting the Nano-Glo Live Cell substrate 20× using Nano-Glo LCS Dilution buffer, and 25 μL was added to each well. Subsequently, the plate was placed in a GloMAX96 plate reader (Promega, Madison, WI, United States). Luminescence was monitored during the equilibration period until the signal stabilized (30 min). For agonist experiments, we added 20 μl per well of test compounds, present as 6.75× stocks in Opti-MEM^®^ I. Also for the analysis of biological extracts, 20 μL was added per well. These extracts were generated from 250 μL of matrix (blood, urine, or vitreous), which was added to 1000 μL of ice-cold acetonitrile, followed by shaking for 5 min at 1400 RPM and centrifuging for 20 min at 20,000 *g*. After evaporation of 1 mL of supernatant under nitrogen at 40°C, the extract was reconstituted in 100 μl of Opti-MEM^®^ I. The luminescence was continuously detected (105 or 120 min).

**FIGURE 2:**
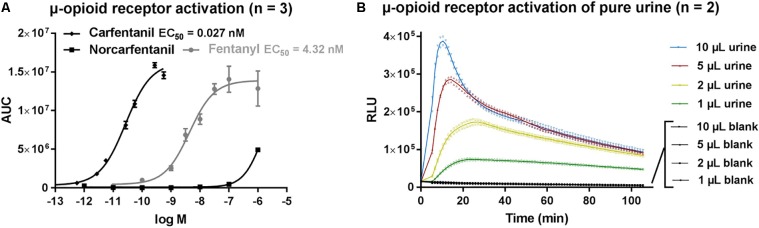
**(A)** μ-opioid receptor activation by fentanyl, carfentanil and norcarfentanil. **(B)** μ-opioid receptor activation of pure urine without sample preparation. AUC, area under curve; RLU, relative light units.

Application of carfentanil and fentanyl solutions on the opioid activity reporter assay resulted in concentration-dependent curves and EC_50_ (95% confidence interval profile likelihood) values were determined for carfentanil [EC_50_ = 0.027 nM (0.021–0.035)] and fentanyl [EC_50_ = 4.32 nM (2.43–7.83)] as a measure of relative potency (**Figure 2A**). Although it is difficult to compare EC_50_ values from different assays (due to different experimental setups), our values are in line with those found in literature. Feasel (2017) stated in his dissertation an EC_50_ of 0.006 nM for carfentanil and 0.511 nM for fentanyl (PerkinElmer^®^ LANCE Ultra cAMP Assay), which supports the significantly stronger potency of carfetanil, as also found here. Norcarfentanil, the major metabolite of carfentanil, was only able to generate low opioid activity at a high concentration (1 μM/326 ng/mL) (**Figure 2A**). All extracts from the three matrices (blood, urine, and vitreous) showed very strong opioid activity. Even application of 1 μL of urine sample from the presented case (without any sample preparation) on the bioassay was able to generate a clearly positive signal, easily distinguishable from negative control blank urine, in the opioid activity reporter assay (**Figure 2B**).

The screening results from the opioid activity reporter assay were confirmed with an LC–MS/MS method for carfentanil and norcarfentanil. To 250 μL sample (blood, urine, or vitreous), 10 μL of internal standard solution containing fentanyl-D_5_ and norcarfentanil-D_5_ (0.25 and 12.5 ng/mL, respectively) in methanol were added. Sample processing was as described above, except that reconstitution was with 55 μL acetonitrile, of which 50 μL were then mixed with 50 μL of mobile phase A (H_2_O + 0.1% HCOOH) in an autosampler vial with 100 μL insert. For the analysis of carfentanil, the injection volume was 20 μL, whereas for the determination of norcarfentanil, 10 μL were injected. Chromatographic separation was achieved on a Kinetex Biphenyl column (50 mm × 2.1 mm, 2.6 μm) (Phenomenex, Utrecht, Netherlands) in a 3.7 min gradient using H_2_O + 0.1% HCOOH and methanol + 0.1% HCOOH as mobile phases, at a flow rate of 0.6 mL/min. The following gradient was used: 0–0.2 min: 5%B, 0.25–0.35 min: 5–30% B, 0.35–1.5 min: 30–95% B, 1.5–2.5 min: 95% B, 2.5–2.51 min: 95–5% B, 2.51–3.7 min: 5% B. A QTRAP 5500 mass spectrometer (SCIEX, Nieuwerkerk aan den Ijssel, Netherlands) with positive electrospray ionization in multiple reaction monitoring mode was used for detection. For carfentanil, the following transitions were used: 395.2 > 246.1 [quantifier, declustering potential (DP): 70 V, collision energy (CE): 27 eV, collision cell exit potential (CXP): 12 V] and 395.2 > 146.2 (qualifier, DP: 70 V, CE: 37 eV, CXP: 9 V). For norcarfentanil, the transitions were 291.1 > 142.2 (quantifier, DP: 74 V, CE: 22 eV, CXP: 7 V) and 291.1 > 146.2 (qualifier, DP: 74 V, CE: 37 eV, CXP: 10 V). For fentanyl-D_5_, 342.2 > 188.2 (DP: 110 V, CE: 32 eV, CXP: 10 V) was used. For norcarfentanil-D_5_, the transition was 296.1 > 151.1 (DP: 75 V, CE: 38 eV, CXP: 8 V). The entrance potential was 10 V for all transitions; source temperature was set to 600°C, ion spray voltage to 2000 V, curtain gas to 35 psi, gas 1 to 40 psi and gas 2 to 50 psi.

The method was validated in whole blood. Eight-point calibration curves were set up for carfentanil (range: 0.0025–2.5 ng/mL, linear regression with 1/x^2^ weighting) and norcarfentanil (range: 0.025–25 ng/mL, linear regression with 1/x^2^ weighting). Quality control samples at 0.015/0.25 ng/mL for carfentanil and at 0.15/2.5 ng/mL for norcarfentanil were run in sixplicate on 4 days, yielding acceptable intra- and inter-run imprecision (intra-run: <8.8%, inter-run: <14%) and bias (< ±8.7%, *n* = 24 at two different concentrations). Matrix effects were assessed at the two above-mentioned concentrations by comparing the signal ratios of analyte to internal standard of post-extraction-spiked samples with those of standards spiked in neat injection solvent (*n* = 6). Matrix effects were 78% for carfentanil and 118% for norcarfentanil. Extraction efficiency, assessed by comparing the signal ratios of analyte to internal standard of pre- versus post-extraction-spiked samples, was 66% for carfentanil and 24% for norcarfentanil (*n* = 6, at the two above-mentioned concentrations). Also, autosampler stability (change in concentration <9% for at least 3 days, *n* = 6, two different concentrations), specificity and carry-over (none within calibration range) were successfully evaluated. Dilution integrity was checked by spiking blood and aqueous samples with 100 ng/mL carfentanil and norcarfentanil, then diluting 1:1000 with blank matrix (*n* = 6) and comparing relative peak areas to control samples with 0.1 ng/mL (*n* = 6). Differences were ≤ ±13.5%.

The vitreous sample was quantified using a calibration curve in ultra-pure water. The urine sample was quantified by standard addition. To quantify carfentanil concentrations, blood and vitreous samples had to be diluted 1:1000 with blank blood and water, respectively, while the urine sample was diluted 1:100 with blank urine. For norcarfentanil, undiluted samples were analyzed. Carfentanil concentrations were 92 ng/mL in blood, 2.8 ng/mL in urine, and 23 ng/mL in vitreous. The blood and vitreous contained 0.532 and 0.300 ng/mL norcarfentanil, respectively. No norcarfentanil was detected in urine. It should be noted that carfentanil concentrations are typically in the sub-ng/mL range (Papsun et al., 2017: 0.1–14 ng/mL, median: 0.38 ng/mL; Shanks and Behonick, 2017: 0.0102–2 ng/mL, median: 0.0984 ng/mL; Hikin et al., 2018: 0.09–4 ng/mL, median: 0.234 ng/mL).

## Discussion

Given the continued emergence of novel synthetic opioids, the major disadvantage for their detection via immunoassays, GC–MS and LC–MS/MS analysis is that the methods are often targeted in nature or, for the latter two, limited by the availability of pre-established mass spectral libraries. Here in this case, the immunoassay for fentanyl did not pick up carfentanil, a fentanyl analog, due to the lack of cross-reactivity. Therefore, an alternative untargeted approach for the detection of (synthetic) opioids, not directly based on the structure of the opioids, but on their opioid activity, was applied. Such an approach may serve as a first-line screening tool, complementing the conventional analytical methods which are currently used.

The high ratio of carfentanil/norcarfentanil in blood and vitreous and the absence of norcarfentanil in urine can be explained by the presumably sudden death of the victim caused by the massive overdose. The detected concentrations of carfentanil are, to the best of our knowledge, the highest ever reported in a human being. Other intoxications always state sub-ng to low ng/mL levels of carfentanil (Müller et al., 2017; Papsun et al., 2017; Shanks and Behonick, 2017; Swanson et al., 2017; Elliott and Hernandez Lopez, 2018; Hikin et al., 2018). In conclusion, this is the first report in which a novel activity-based opioid screening assay was successfully deployed in a forensic case, where confirmation and quantification using a validated bioanalytical procedure revealed very high carfentanil concentrations.

## Ethics Statement

We received permission from the Belgian Department of Justice to use the samples for this study.

## Author Contributions

AC was involved in the development and application of the bioassay and wrote the manuscript. LA worked on the development and validation of the LC–MS/MS method and wrote the manuscript. PB provided the carfentanil standard, gave additional information concerning the carfentanil package found at the scene, and checked the final version of the manuscript. CS was the forensic toxicologist in charge of the case and wrote the manuscript.

## Conflict of Interest Statement

The authors declare that the research was conducted in the absence of any commercial or financial relationships that could be construed as a potential conflict of interest.
